# Alexithymia and Impaired Mentalization: Evidence from Self-, Informant-, and Meta-Perception Ratings on the 20-Item Toronto Alexithymia Scale

**DOI:** 10.3390/jintelligence13070089

**Published:** 2025-07-21

**Authors:** R. Michael Bagby, Luigia Zito, Sharlane C. L. Lau, Ardeshir Mortezaei, Piero Porcelli, Graeme J. Taylor

**Affiliations:** 1 Department of Psychology, University of Toronto, Toronto, ON M5S 2E5, Canada; sharlane.lau@mail.utoronto.ca; 2 Department of Psychiatry, University of Toronto, Toronto, ON M5T 1R8, Canada; graeme.taylor@utoronto.ca; 3 Graduate Department of Psychological Clinical Sciences, University of Toronto, Toronto, ON M1C 1A4, Canada; 4 Department of Psychological, Humanistic and Territorial Sciences, University G. d’Annunzio of Chieti-Pescara, 66013 Chieti, Italy; gina.zito@tiscali.it (L.Z.); piero.porcelli@unich.it (P.P.); 5 Department of Psychology, Ontario Tech University, Oshawa, ON L1G 0C5, Canada; ardeshir.mortezaei@ontariotechu.net

**Keywords:** alexithymia, mentalization, meta-perception, meta-mentalization, emotional intelligence, TAS-20, multi-informant assessment, reflective functioning, theory of mind, emotional awareness

## Abstract

Alexithymia is a trait-like deficit in the cognitive processing of emotions, characterized by difficulty identifying and describing feelings, externally oriented thinking, and limited imaginal capacity. It reflects a deficit in emotional intelligence, specifically in the intrapersonal ability to understand and manage one’s own emotional states and to similarly recognize how others might view them. Emotional intelligence has been conceptualized as a distinct form of intelligence that involves emotion-related mental abilities and meets standard psychometric criteria for inclusion within the broader taxonomy of human intelligences. Increasingly, alexithymia is also understood as a failure of affect-focused mentalization, or the ability to perceive emotions in oneself and others as intentional states. This study examined alexithymia using a multi-informant approach to assess intrapersonal and interpersonal emotional awareness. A sample of 211 university students and their informants completed the Toronto Alexithymia Scale (TAS-20), an informant version (TAS-20-IF), and a novel meta-perception version (TAS-20-Meta). Two hypotheses were tested and supported: (1) participants underestimated their alexithymia traits relative to informant ratings and (2) self- and meta-perception ratings were more strongly correlated than either was with informant ratings. These findings support the view that alexithymia reflects deficits in both affective mentalization and a specific domain of human intelligence.

## 1. Introduction

Alexithymia reflects, in part, a deficit in emotional intelligence (EI), particularly in the intrapersonal capacities to perceive, understand, and regulate one’s own emotional states and to appreciate how others perceive them (Mayer and Salovey 1993; Mayer et al. 1999; Taylor et al. 1997). EI, in turn, has been proposed as a legitimate form of intelligence within the broader cognitive science literature: it involves mental abilities that are distinct from, but related to, general intelligence, and it meets classical psychometric criteria for the construct of intelligence, including developmental progression, coherent internal structure, and real-world utility (Mayer et al. 1999, 2008). From this perspective, alexithymia can be conceptualized as a trait-like impairment in a specific domain of human intelligence; namely, the ability to perceive, understand, and reason about emotional information. Alexithymia reflects an inverse of EI, representing a developmental and functional limitation in emotion-related reasoning capacities that are foundational to adaptive self-awareness and interpersonal understanding.

Most generally, EI refers to the adaptive capacity to perceive, use, understand, and regulate emotions in oneself and others (Mayer et al. 2016; Mayer and Salovey 1997), whereas alexithymia refers to a trait-like deficit in the cognitive processing of emotions, particularly involving difficulty identifying and describing one’s feelings, as well as an externally oriented mode of thinking and a limited imaginal capacity (Luminet and Nielson 2025; Taylor et al. 1997). These two constructs appear to reflect opposite poles on a continuum of emotional self-awareness: individuals with high levels of EI typically demonstrate greater access to their emotional experience and better interpersonal functioning, while individuals with high levels of alexithymia often experience poor emotional awareness and an impaired capacity to regulate and communicate emotions effectively (Parker et al. 2001). Studies have found that the self-reported 20-item Toronto Alexithymia Scale (TAS-20; Bagby et al. 2020) correlates negatively with both self-reported and performance-based measures of EI (Lumley et al. 2005; Mikolajczak et al. 2007; Parker et al. 2001).

Despite their inverse relationship, alexithymia and EI have only occasionally been considered together in integrative models of emotional functioning. Both can be understood, however, as reflecting broader capacities for reflective functioning, or the ability to mentally represent emotional states in oneself and others. This perspective aligns with Lane and Schwartz’s (1987) cognitive development model, which posits that the ability to identify and articulate emotions develops through hierarchical levels of cognitive representational complexity (Lane and Schwartz 1987). In recent years, scholars have increasingly turned to mentalization theory to bridge these constructs, framing alexithymia as a failure in affect-focused mentalizing—that is, in the capacity to interpret one’s own and others’ emotional states as meaningful and intentional (Fonagy and Luyten 2009; Jurist 2005). Within this framework, mentalization offers a higher-order account of intrapersonal EI (the ability to access one’s own emotional life) and interpersonal EI (i.e., the ability to read the moods, intentions, and desires of others) (Gardner 1983), integrating affect recognition, emotion regulation, and theory of mind into a single developmental and neurocognitive system (Bateman and Fonagy 2016). Alexithymia, in turn, can be conceptualized as a disruption in this system, particularly in its self-focused dimension, which impairs an individual’s ability to identify and understand their own emotions and navigate emotional situations effectively.

In contrast, mentalization is a multidimensional construct that is related closely not only to alexithymia and EI, but also to empathy, mindfulness, theory of mind, psychological mindedness, and insight (Fonagy et al. 2012). And whereas mentalization pertains to the full range of mental states, alexithymia and EI are restricted to emotional mental states; empathy is the awareness of others’ emotions and thoughts, and one’s responsiveness to them; psychological mindedness has been construed as the disposition to mentalize; and insights are generated by skillful mentalizing (Allen et al. 2008). There is also neuroscientific evidence indicating that alexithymia is associated with disruptions in the neural systems underlying empathy, further linking emotional self-awareness to interpersonal mentalizing (Bird and Viding 2014).

In addition to these overlapping constructs, an emerging literature on meta-perception, construed as one’s beliefs about how others perceive oneself, is providing a novel way to operationalize a facet of mentalization in interpersonal terms (Carlson and Barranti 2016; Carlson and Kenny 2012; Human and Biesanz 2013). Described by some researchers as a form of “second-order mentalizing,” meta-perception (also referred to as “meta-mentalization”) connects self-focused and other-focused emotional understanding (Wu et al. 2020, 2022). Its integration into alexithymia research allows for a more dynamic and nuanced model of socio-emotional functioning that aligns with the broader aims of intelligence research, particularly in how individuals interact and relate with others emotionally. Individuals tend to be preoccupied with how they are perceived by others (Grutterink and Meister 2021; Kenny 1988; Sheldon and Johnson 1993). In their daily social relationships, individuals interpret and decipher the thoughts and judgments of others, leading to the development of meta-perceptions (Grutterink and Meister 2021; Kenny 1988). Meta-perceptions influence EI and guide critical life choices in behavior, friendships, professional relationships, and romantic pursuits (Carlson and Barranti 2016). These perceptions are integral to how people navigate complex social and socio-emotional environments. However, there is a key concern regarding the accuracy of meta-perceptions (meta-accuracy) (Carlson and Furr 2009, 2011; Kenny and DePaulo 1993). Meta-perceptions may also differ across social groups or contexts, as shown in early interpersonal perception studies (Malloy et al. 1997). Whereas individuals often hold a fairly accurate general understanding of the impressions they make on others, people with maladaptive personality traits may have difficulty comprehending how others view them (Kenny and DePaulo 1993; Oltmanns et al. 2005).

Research on meta-perception in individuals with maladaptive personality traits suggests that they may have a poor understanding of themselves and make incorrect assumptions about how others experience them (Carlson and Oltmanns 2015). This gap in self-awareness can lead to a distorted view of their emotional functioning, making it more challenging to improve EI and interpersonal relationships. Exploring meta-perception in relation to alexithymia allows for a better understanding of the interpersonal manifestations of alexithymia and its associations with key dimensions of EI, such as affect recognition, emotion regulation, and social insight. Bridging the gap between self-focused emotional awareness and interpersonal emotional understanding can contribute to a more dynamic model of emotional functioning that considers both the intrapersonal and interpersonal aspects of EI.

## 2. The Present Study

In the present study we examine the relationship between alexithymia and an aspect of mentalization (vis., understanding how others perceive one’s own mental states). We operationalized this aspect of mentalization through the construct of meta-perception. Specifically, we analyzed three perspectives: (1) self-report—i.e., how “targets” rate themselves on the TAS-20; (2) informant-report—i.e., how informants rate the target on the TAS-20-IF; and (3) meta-perception—i.e., how targets believe their informants would rate them on the TAS-20-IF completed by the target from the informant’s imagined perspective. By comparing these perspectives, we aimed to assess the extent to which individuals with varying levels of alexithymia show concordance between self-perception, perceived other-perception, and actual other-perception.

### Hypotheses

Consistent with theoretical and empirical literature on some of the shared features of mentalization, alexithymia, and EI (see Moriguchi et al. 2006; Parker et al. 2001; Rosso 2022; Rothschild-Yakar et al. 2019; Taylor and Bagby 2013), we conceptualize alexithymia as involving deficits in core components of EI and mentalization and proposed and tested two hypotheses: (1) the intrapersonal deficit hypothesis; here we hypothesized that there will be a significant difference between self-report (TAS-20) and informant report (TAS-20-IF) total scores, with self-report scores being lower than informant report scores. This discrepancy reflects a deficit in intrapersonal EI, manifesting as reduced emotional self-awareness; (2) the interpersonal deficit hypothesis; here we hypothesized a specific pattern of correlations and mean score differences and correlations among and between the TAS-20, TAS-20-IF, and TAS-20-Meta scores. Specifically, we predicted that (a) TAS-20 self-report scores would be more strongly correlated with TAS-20-Meta (meta-perception) scores than with TAS-20-IF (informant) scores, reflecting a projection of self-view rather than accurate inference of others’ perspectives, and (b) TAS-20-IF scores would be significantly higher than TAS-20-Meta scores, indicating that individuals underestimate how alexithymic they are perceived to be by others. Together, this pattern of results would reflect a deficit in interpersonal EI—a limited ability to accurately infer how others perceive one’s emotional functioning (a core capacity in both EI and mentalization). These effects would indicate a failure of interpersonal mentalization, or second-order mentalizing, which is a core component of interpersonal EI.

## 3. Methods

### 3.1. Participants

The data used in the present study were obtained from an archival database, and some of the data (i.e., TAS-20 and TAS-20-IF ratings) have been previously published in Bagby et al. (2021), which provides a detailed description of the recruitment and data collection procedures. Briefly, each participant (target) completed the TAS-20 to assess self-perceived alexithymia and nominated informants to complete the TAS-20-IF. Targets also completed the TAS-20 meta-perception version (TAS-20-Meta).

The archived dataset comprised test protocols from 243 undergraduate students at a large Canadian university. For the purpose of this study, we included test protocols of students who completed the TAS-20 and for whom we were able to match their TAS-20 with TAS-20-IF and TAS-20-Meta^1^. The final sample used for analyses included 211 undergraduate students (56 males, 155 females [73.5%]). The mean reported age of the sample is 18.82 (*SD* = 1.91; range = 18–38). The self-reported ethno-racial composition of the target sample was as follows: 46 South Asian (21.8%), 41 East Asian (19.4%), 37 Southeast Asian (17.5%), 28 White/European (13.2%), 15 West Indian (7.1%), 13 Middle Eastern (6.2%), 9 multi-racial (4.3%), 8 Black (3.8%), 4 Latin American (1.9%), and 10 (4.7%) reported having other ethno-racial identities or preferred not to answer.

The informant sample included 77 males and 134 females (63.5%), and the mean reported age is 24.94 (*SD* = 12.15; range = 14–62). The self-reported ethno-racial composition of the informant sample was as follows: 44 South Asian (20.9%), 40 East Asian (19.0%), 36 White/European (17.1%), 31 Southeast Asian (14.7%), 15 Black (7.1%), 14 Middle Eastern (6.6%), 12 West Indian (5.7%), 8 multi-racial (3.8%), 1 Latin American (0.5%), and 10 (4.7%) reported having other ethno-racial identities or preferred not to answer. Informants had known the targets for at least one year. They were asked to rate how close they were with the target on a scale of 0 (not at all close) to 10 (extremely close), and the mean rating was 8.76 (*SD* = 1.55). Most informants were in frequent contact with the target participant, with 57.3% (*n* = 121) reporting that they spoke with or saw the nominator every day, 19.9% (*n* = 42) reporting having contact with the target 3–4 times per week, and 19.0% (*n* = 40) reporting they had contact with the target 1–2 times per week. A small proportion (3.3%; *n* = 7) reported having no contact with the target^2^.

### 3.2. Measures

#### 3.2.1. 20-Item Toronto Alexithymia Scale (TAS-20) (Self-Report)

The TAS-20 (Bagby et al. 1994) is a 20-item self-report scale assessing alexithymia and is the most frequently and widely used measure of the alexithymia construct. It has been employed in hundreds of studies over the past 25 years (Bagby et al. 2020). The scale was developed to operationalize the alexithymia construct as originally formulated by Nemiah and Sifneos (1970) and Nemiah et al. (1976) and later elaborated by Taylor et al. (1997). The 20 items are rated on a 5-point Likert scale ranging from 1 (strongly disagree) to 5 (strongly agree). The scale yields a total alexithymia score and can also be divided into three factor subscales: Difficulty Identifying Feelings (DIF), Difficulty Describing Feelings (DDF), and Externally Oriented Thinking (EOT). Although some researchers report subscale scores, the scale developers generally recommend using the total score as the most reliable and valid indicator of overall alexithymia (Bagby et al. 2020). The TAS-20 has demonstrated strong psychometric properties, including internal consistency, test–retest reliability, and construct and predictive validity. Its three-factor structure is stable and replicable, as evidenced by a recent meta-analytic study (Schroeders et al. 2022).

#### 3.2.2. 20-Item Toronto Alexithymia Scale-20 Informant Form (TAS-20-IF) (Informant-Report)

The TAS-20-IF (Bagby et al. 2021) is the informant version of the self-report TAS-20. The TAS-20-IF includes male, female, and gender-neutral versions.^3^ The TAS-20-IF retains the three-factor structure of the TAS-20 (DIF, DDF, EOT). The TAS-20-IF demonstrated adequate internal consistency and a factor structure that closely mirrored that of the TAS-20, indicating that both instruments assess the same underlying construct.

#### 3.2.3. 20-Item Toronto Alexithymia Scale (TAS-20-Meta) (Meta-Perception Version)

The TAS-20-Meta is not a formal scale per se, but rather a variable created for this study to assess the targets’ perception of how their informants would rate the targets’ level of alexithymia. The item content and format correspond to the TAS-20, but respondents are instructed to answer based on how they think their nominated informant would rate them based on each item. Some of the items were slightly reworded from the self-report TAS-20 to clarify this instruction; for example, Item 5, “I am able to describe my feelings easily”, was changed to “Close others would say that I am able to describe my feelings easily.”

### 3.3. Statistical Analysis

Coefficient alphas were computed to examine the internal consistency of the total scores of the TAS-20, TAS-20-IF, and TAS-20-Meta. Descriptive statistics were computed for each of the three alexithymia ratings. The mean differences between scores across ratings were analyzed using pairwise, non-independent *t*-tests, supplemented with Cohen’s *d* effect size estimates, which were interpreted using standard benchmarks in which 0.20 indicates a small effect, 0.50 a medium effect, and 0.80 a large effect (Cohen 1988; Sawilowsky 2009).

Intraclass correlations (ICCs) were used to assess the associations between pairs of ratings. Although, as indicated earlier, Bagby et al. (2020) generally recommend using only the total TAS-20 score to assess alexithymia, we chose to examine correlations between total and subscale scores across self-report, informant-report, and meta-perception measures to explore which facets of alexithymia demonstrated greater concordance across rating sources. Including these correlations provided more nuanced information about which facets of alexithymia may be more or less observable to others and more or less accurately perceived by the targets themselves. A two-way mixed ICC model with absolute agreement definition and single measures was selected. The two-way mixed model was chosen as the same set of raters (i.e., targets and their informants) evaluated each target, and these raters were not randomly sampled from a larger population. The absolute agreement definition was selected to examine the degree to which target participants’ self-perceptions matched the absolute scores from informants or their own meta-perceptions. Single-measure ICCs were reported, as each rating perspective involved a single rating per target rather than aggregated ratings (Koo and Li 2016). ICCs were interpreted according to established guidelines (Koo and Li 2016), with values < 0.50 considered poor, 0.50–0.75 moderate, 0.75–0.90 good, and >0.90 excellent. We compared the 95% confidence intervals (CIs) of ICCs to assess differences between correlations. A lack of overlap between the 95% CIs indicates a significant difference at the 5% significance level. All statistical analyses were conducted using IBM SPSS Statistics Version 29.0.1.1.

## 4. Results

**Figure 1:**
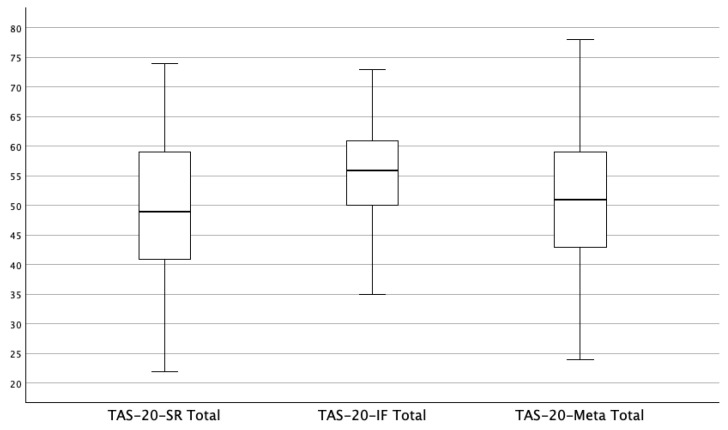
Boxplot of TAS-20-SR, TAS-20-IF, and TAS-20-Meta total scores. Note: TAS-20-SR = Toronto Alexithymia Scale Self-Report; TAS-20-IF = Toronto Alexithymia Scale Informant Form; TAS-20-Meta = Toronto Alexithymia Scale Meta-Perception Version.

The TAS-20, TAS-20-IF, and TAS-20-Meta evidenced good internal consistency, with the coefficient alpha values for the total scores of 0.83, 0.84, and 0.82, respectively.^4^ The results of the paired *t*-tests comparing self-report, informant, and meta-perception TAS-20 total score ratings are displayed in Table 1. A boxplot illustrating the distribution of TAS-20 total scores across the three rating perspectives is provided in Figure 1. The ICCs among target, informant, and meta-perception TAS-20 total scores (and subscale scores) are displayed in Table 2. These results support both the intrapersonal and interpersonal deficit hypotheses. For the intrapersonal hypotheses, as predicted, the TAS-20 total score was significantly lower than the TAS-20-IF total score. For the interpersonal hypothesis, as predicted, the TAS-20-IF total score was significantly higher than the TAS-20-Meta total score, and as determined by the magnitude of the ICC coefficients (see Koo and Li 2016), there was also a strong or good level of agreement between self-report and meta-perception total ratings, which was in turn significantly different from the weak or poor level of agreement between meta-perception and informant ratings. This pattern of correlations was consistent across TAS-20 factor subscales, with the exception of the EOT factor subscale, where the correlation between self-report and meta-perception ratings was moderate but still significantly higher than the correlation between meta-perception and informant ratings. This was likely attributable to the lower coefficient alpha of this factor subscale, which is attributable to its multidimensional nature. It is important to note that the developer of the TAS-20 recommends only the use of a total score; in this study we only used the subscales to provide preliminary insights into which components of alexithymia contributed to agreement across the TAS-20, TAS-20-IF, and TAS-20-Meta. 

Although we generated no hypotheses regarding whether familiarity or frequency of interpersonal contact influenced the discrepancies between TAS-20 self-report and informant ratings. Spearman’s rho correlations indicated that neither familiarity (ρ = 0.007) nor frequency of contact (ρ = −0.022) was significantly associated with discrepancies (both *p* > 0.75).

## 5. Discussion

The present findings support the conceptual association between alexithymia and impaired mentalization. Target participants, on average, underestimated their own difficulties with emotional awareness and style of thinking relative to informant perceptions and showed limited insight into how others perceive them; these results suggest reduced capacity for meta-perception. Reduced capacity for meta-perception supports, in part, the contention that alexithymia is a trait characterized by diminished overall emotional self-awareness. This lack of emotional self-awareness and understanding of others’ perspectives highlights the broader implications of alexithymia, which affects both self- and other-related mentalization processes. The significant and medium effect size correlation between the EOT subscale of the TAS-20-Meta with the TAS-20-SR, but a nonsignificant small effect correlation with the TAS-20-IF, is consistent with the view that the operative thinking (*pensée opératoire*) component of the alexithymia construct contributes to a deficiency in mentalizing (Taylor et al. 2023).

Meta-perception (or meta-mentalization), defined as the ability to understand how others perceive oneself, plays a crucial role in social functioning (Wu et al. 2020, 2022). Impairments in meta-perception observed in individuals with alexithymia may help explain the challenges they face in interpersonal interactions and their difficulties in establishing and maintaining effective relationships. There is evidence from other research that individuals with high levels of alexithymia tend to be indifferent and distant in their interpersonal relationships, refrain from sharing their own needs and desires, express little affection, and negatively influence the satisfaction of their marital or romantic partners (Hesse and Floyd 2008; Humphreys et al. 2009; Vanheule et al. 2007; Yelsma and Marrow 2003). These limitations are generally attributed to poor emotional self-awareness, lack of empathy, and low EI. However, the finding of a strong correlation between self-report and meta-perception, coupled with the weak correlation between meta-perception and actual informant ratings, suggests that individuals with alexithymia may also project their own self-view onto others rather than accurately discerning external perspectives.

The results from our study underscore the importance of incorporating both self- and informant-report measures in the assessment of alexithymia. Exclusive reliance on self-report may obscure deficits that are more observable to others, particularly in individuals with poor emotional insight. The multi-informant approach, combining self-reports, informant ratings, and structured interviews, provides a comprehensive, multi-informant perspective on alexithymia traits. Most broadly, evidence suggests that self-other discrepancies are systematic and that informant reports can provide incremental information beyond self-report when self-awareness is impaired (Kim et al. 2019; Olino and Klein 2017). For the assessment of alexithymia, the concurrent use of the TAS-20 and TAS-20-IF enables the examination of both how an individual perceives themselves and how others perceive them, thereby addressing potential discrepancies in self-perception. Additionally, structured interviews, such as the Toronto Structured Interview for Alexithymia (TSIA; Bagby et al. 2006), offer a clinician-guided middle ground, enhancing reliability and depth through behavioral probing.

The implications for treatment, particularly with mentalization-based therapies (MBT), are noteworthy. MBT focuses on enhancing reflective functioning, which involves the ability to understand one’s own and others’ mental states, especially in the context of emotional relationships (Luyten et al. 2020). Since alexithymia reflects a limitation in both self- and other-related emotional awareness, individuals with higher levels of alexithymia may benefit from interventions that specifically target these capacities (Löf et al. 2018). For example, in a study of patients with major depressive disorder, Bressi et al. (2017) reported an inverse relationship between alexithymia and reflective functioning and that depression and TAS-20 scores improved in response to short-term psychodynamic psychotherapy that incorporated mentalization-based techniques. MBT aims to improve an individual’s ability to mentalize, or reflect on mental states as they occur, and through the rebalancing of mentalizing, it fosters a more nuanced understanding of self and others. This, in turn, can potentially improve social functioning and reduce maladaptive behaviors.

The central aspect or presumption of the MBT therapeutic model is that difficulties in affect regulation are thought to stem from problems in mentalization. MBT is designed to assist patients in better identifying their feelings and directing their thinking toward their internal states (Löf et al. 2018). The use of tools like the TAS-20, TAS-20-IF, and TSIA in pre- and post-treatment assessments can help track therapeutic progress. Improvements in the congruence between self-reports and informant reports may serve as an indicator of enhanced mentalization and insight, reflecting the potential gains in emotional awareness and understanding. This approach aligns with recent evidence showing specific profiles of impaired self- and other-oriented mentalization in individuals with eating disorders, particularly bulimia (Telléus et al. 2024). Future research should aim to broaden the scope of this approach by applying it to diverse clinical populations, particularly those with a range of different mental disorders. A key area of exploration would be whether improvements in meta-perception, as measured by greater alignment between self-, informant, and meta-perception reports, are predictive of broader treatment outcomes.

Researchers should investigate whether these improvements could serve as reliable markers for the effectiveness of therapeutic interventions across various conditions. Moreover, understanding the relationship between enhanced meta-perception and key therapeutic outcomes could offer valuable insights into the underlying mechanisms of change within treatments such as mentalization-based therapies (MBT). Incorporating clinical assessment instruments such as the TAS-20, TAS-20-IF, and TSIA into clinical practice could significantly contribute to this effort. By utilizing these instruments together, clinicians could gain a more comprehensive and accurate evaluation of a patient’s emotional perception. This integrated approach would not only enhance the diagnostic process, providing a more nuanced understanding of the patient’s emotional and psychological functioning, but also allow for more precise and personalized treatment planning. As a result, clinicians would be better equipped to optimize treatment strategies tailored to the individual needs of each patient. Additionally, longitudinal studies exploring the link between meta-perception and treatment outcomes would offer a deeper understanding of the long-term benefits of improving emotional awareness, including sustained treatment success and improved quality of life for patients.

Despite several strengths of the current study, including the incorporation of self-, informant-, and meta-perception ratings and the application of analytic methods to test several key aspects of the original or affect deficit model of alexithymia, several limitations should be noted. First, the mean TAS-20 score in the target sample was relatively low, but typical of university student samples and may have limited the variability needed to detect stronger effects, compromising its generalizability to more severely affected populations. Second, the target sample was composed exclusively of university students, which may not reflect the broader population, particularly clinical groups in which alexithymia is known to be elevated. Third, although it was a requirement for the targets and their informants to have known each other for at least one year, the actual duration of their relationships was not determined. Knowing if the relationships were of much longer duration would increase confidence that informants were better able to provide meaningful ratings of their targets. Previous studies suggest that differences between self- and other-perception are also influenced by the types of personality traits or behaviors being judged (Vazire 2010). With regard to alexithymia, clinicians have long reported that individuals with this trait are usually unaware of their deficits and are often bewildered when someone close to them (such as a spouse) complains of the lack of emotional communication (e.g., Swiller 1988).

Although there is extensive research supporting the validity of the TAS-20 (Bagby et al. 2020), this scale has been criticized for some possible shortcomings, including a failure to assess awareness of positive emotions, an overlap with measures of negative affect, and low reliability of the EOT subscale (Kooiman et al. 2002; Preece et al. 2020; Subic-Wrana et al. 2005). For these and other reasons, the developers of the scale have always recommended that it be used as a screening instrument for alexithymia and part of a multi-method measurement assessment approach (Taylor et al. 1997). Future studies should aim to replicate our findings in clinical and community-based samples with a wider range of alexithymia severity and should consider including structured interviews (e.g., TSIA) and performance-based measures of emotional insight to enhance construct validity. Despite the above limitations, the study contributes novel evidence supporting the interpersonal and intrapersonal deficits associated with alexithymia and underscores the value of integrating meta-perception measures into alexithymia research.

## Figures and Tables

**Table 1:** Paired *t*-tests comparing TAS-20-SR, TAS-20-IF, and TAS-20-Meta total ratings.

**Descriptive Statistics**						
	Mean	*SD*				
TAS-20-SR	49.18	11.66				
TAS-20-IF	55.63	8.61				
TAS-20-Meta	50.56	11.41				
**Paired *t*-tests**						
	Paired differences					
	Mean	*SD*	*t*	df	*p*	Cohen’s *d*
TAS-20-SR TAS-20-IF	−6.47	13.17	−7.14	210	<0.001	−0.49
TAS-20-SR TAS-20-Meta	−1.40	7.24	−2.81	210	0.005	−0.19
TAS-20-IF TAS-20-Meta	5.07	12.92	5.70	210	<0.001	0.39

Note: TAS-20-SR = Toronto Alexithymia Scale Self-Report; TAS-20-IF = Toronto Alexithymia Scale Informant Form; TAS-20-Meta = Toronto Alexithymia Scale Meta-Perception Version.

**Table 2:** Intraclass correlations among target, informant, meta-perception TAS-20 total and subscale scores.

**Total Scale**		
	TAS-20-SR Total	TAS-20-IF Total
TAS-20-SR Total	--	--
TAS-20-IF Total	0.15 ** (0.02, 0.27)	--
TAS-20-Meta Total	0.80 *** (0.74, 0.84)	0.16 ** (0.03, 0.29)
**DIF**		
	TAS-20-SR DIF	TAS-20-IF DIF
TAS-20-SR DIF	--	--
TAS-20-IF DIF	0.23 *** (0.10, 0.36)	--
TAS-20-Meta DIF	0.84 *** (0.79, 0.87)	0.23 *** (0.10, 0.35)
**DDF**		
	TAS-20-SR DDF	TAS-20-IF DDF
TAS-20-SR DDF	--	--
TAS-20-IF DDF	0.12 * (−0.01, 0.25)	--
TAS-20-Meta DDF	0.75 *** (0.69, 0.81)	0.16 * (0.02, 0.28)
**EOT**		
	TAS-20-SR EOT	TAS-20-IF EOT
TAS-20-SR EOT	--	--
TAS-20-IF EOT	−0.02 (−0.07, 0.04)	--
TAS-20-Meta EOT	0.54 *** (0.35, 0.68)	0.01 (−0.05, 0.08)

Note: * *p* < 0.05, ** *p* < 0.01, *** *p* < 0.001. The 95% confidence intervals are displayed in parentheses. TAS-20-SR = Toronto Alexithymia Scale Self-Report; TAS-20-IF = Toronto Alexithymia Scale Informant Form; TAS-20-Meta = Toronto Alexithymia Scale Meta-Perception Version.

## Data Availability

The data presented in this study are available on request from the corresponding author due to restrictions related to Research Ethics Board requirements and the lack of participant consent to share the data in a publicly accessible repository.

## Notes

1 Although the same general dataset was used in this investigation as employed by Bagby et al. (2021), the sample sizes across studies differ, as the earlier study did not include TAS-20-Meta ratings.

2 These target and informant sample sizes and ethno-racial composition differ from Bagby et al. (2021) in that this earlier investigation did not include matching TAS-20-Meta data with TAS-20 (target) and TAS-20-IF (informant), which resulted in a smaller sample size in the current study.

3 In this study we used the male/female version.

4 The corresponding alpha coefficients for the factor subscales were as follows: TAS-20—DIF (0.85), DDF (0.73), EOT (0.49); TAS-20-Meta—DIF (0.86), DDF (0.78), EOT (0.49); TAS-20-IF—DIF (0.79), DDF (0.72), EOT (0.35); note the EOT values are lower due to the multidimensional nature of this subfactor.
